# Division Tendo-Achilis—Silver Suture

**Published:** 1874-09

**Authors:** 


*MEDICAL RECORD—May,* 1874:

Division of Tendo-Achillis—Silver Sutures.— * * * The patient, Jos. E. R., age fifty, publisher, 59 Beekman street, was present by invitation. On Friday evening, January 9th, while bathing at French's Hotel, thrust his foot through the porcelain basin of a stationary washstand, and the sharp edge cut through the skin, fascia and tendon, followed by copious arterial hemorrhage. This latter was speedily controlled by torsion and styptics, and the lower cut end was observed standing out in bold relief, the surrounding skin retracted, and to the touch feeling like a painter's brush, the interspace between the extremities being about three inches.

By a splint firmly fixed on anterior aspect of the leg and below the knee, the foot was extended. Strips of adhesive plaster were then applied over the calf, crossing like a pair of suspenders, and secured under the plantar arch; the edges of the wound brought together with two stitches, the limb placed on an *inclined* plane, and the whole treatment continued thus, and completed with ice-cold water dressings.

The fifth day the patient rises on a cane, and the seventh, in a shoe with high counter, an initial bandage and woollen sock, attends to business and other duties, in his carriage, and is to-day able to walk, with the help of a cane, a short distance.

The gastrocnemius and soleus, parent muscles of this tendon, are noticeably quiet, any effort on their part in the lifting of the heel, even poising the body on the injured foot, being instantly followed by the patient quickly tottering to fall.

*Queries.*—On this thirteenth day after the injury, is this good result exceptional? and what would be the result of bringing the cut ends together by silver sutures?

Dr. O'Sullivan considers this case remarkable in result, also rare in occurrence; would have prognozed a year.

Dr. Abbott saw a case of a woman in whom the tendon was cut by a broken edge of a chamber vessel; locomotion in about twelve months. This result is remarkable.

Dr. Skiff considers the suture has not a precedent. Had a case of a lad thirteen years of age, cut by a mower; was under observation about a year, and at the end of that time walked tolerably well. A second case of an old lady, frightened, stepping hastily from her carriage, ruptured this tendon; saw her about a year afterwards, and she walked round pretty well.

Dr. Garrish thinks that the suture thus employed should be silver, or other metal. The present result excellent.

Dr. Mulreany never had a case of injury to tendo-achillis of like character. Heard of a case, while in Ireland, of a man cutting the tendon with a shearing-knife, while harvesting sea-weed. No other assistance at hand, a shoe-maker sewed up the wound; in a few months the one foot was as strong as the other; considers twelve months remarkable to secure good result.

REMARKS BY THE EDITOR ATLANTA MEDICAL AND SURGICAL JOURNAL.—So obvious would seem to be the propriety of bringing together the severed extremities of a tendon divided by incision or rupture, with silver wire sutures, that the writer did this as far back as 1861, without supposing for a moment that he had done anything new, or, indeed, anything which would not readily suggest itself to an intelligent mind.

Case.—Sam, negro, aged twenty-five, carpenter, was brought into my office on the 16th of May, 1861, with complete division of the tendo-achillis of the right leg. He boasted of his dexterity in laying down flooring, in the old-fashioned way, by adzing off the inequalities of the plank so as to bring them to a level. It was his practice, after using the adze in front, to throw it over behind him, with a partial rotation of the body, and "cut out the chip" without changing his footing. Whilst thus engaged, and "running a race" with a fellow workman, his adze slipped upon the plank in making the backward lick, and entered the posterior surface of the right tibia, severing all the tissues behind it.

He was placed upon a lounge, the wound examined, and an interval of quite three inches found to intervene between the severed ends of the tendon. A farmer, Mr. Roland Bryant, who chanced to be in the office at the time, was directed to hold the cut extremities in contact, after I had seized the upper fragment with a strong vulsullum passed up into the sheath, and drawn it down, whilst I connected them firmly with two strong sutures of silver wire. There was good command of the foot at once, and the union of the tendon was apparently perfect in two weeks time.

Neither Erichsen nor Druitt speak of the suture in any form as a remedy for severed or ruptured tendons, but confine their injunctions to rest and relaxation of the muscles concerned. In Holmes' System of Surgery we learn: "The mode of bringing the divided ends of the tendon together by means of sutures can scarcely be recommended, as they produce irritation, and keep the wound open. Bandages are not well borne; rest, therefore, with position, must mostly be trusted for the cure." Turning, now, to the great work of Prof. Gross, we find the silver suture fully enjoined: "The cut ends must be carefully approximated by the silver suture, the rest of the wound being firmly closed; or the limb with which the tendon is connected must be placed in the most thoroughly relaxed position possible, in order to approximate its extremities, and thus afford them an opportunity of reuniting; an occurrence, however, which, I am sure, will rarely take place under any circumstances, however propitious. The experiments of Dr. Sivert, of Mobile, performed many years ago, prove that union between the divided ends of a tendon will be much more likely to proceed kindly and satisfactorily, when the parts are held in contact by a metallic suture than by an ordinary one. My opinion is that practitioners have not profited enough by the results of these researches."

